# Fetal Alcohol Spectrum Disorder: The Honey Bee as a Social Animal Model

**DOI:** 10.3390/life14040434

**Published:** 2024-03-25

**Authors:** Marcelo P. Camilli, Olena M. Simko, Breanne Bevelander, Jenna M. Thebeau, Fatima Masood, Marina C. Bezerra da Silva, Muhammad Fahim Raza, Sofiia Markova, Oleksii Obshta, Midhun S. Jose, Sarah Biganski, Ivanna V. Kozii, Michael W. Zabrodski, Igor Moshynskyy, Elemir Simko, Sarah C. Wood

**Affiliations:** 1 Department of Veterinary Pathology, Western College of Veterinary Medicine, Saskatoon, SK S7N 5B4, Canada; marcelo.camilli@usask.ca (M.P.C.);; 2 Department of Veterinary Microbiology, Western College of Veterinary Medicine, Saskatoon, SK S7N 5B4, Canada; 3 Prairie Diagnostic Services Inc., Saskatoon, SK S7N 5B4, Canada

**Keywords:** animal models of human disease, fetal alcohol spectrum disorder, honey bee

## Abstract

Animal models have been essential for advancing research of fetal alcohol spectrum disorder (FASD) in humans, but few animal species effectively replicate the behavioural and clinical signs of FASD. The honey bee (*Apis mellifera*) is a previously unexplored research model for FASD that offers the distinct benefit of highly social behaviour. In this study, we chronically exposed honey bee larvae to incremental concentrations of 0, 3, 6, and 10% ethanol in the larval diet using an in vitro rearing protocol and measured developmental time and survival to adult eclosion, as well as body weight and motor activity of newly emerged adult bees. Larvae reared on 6 and 10% dietary ethanol demonstrated significant, dose-responsive delays to pupation and decreased survival and adult body weight. All ethanol-reared adults showed significantly decreased motor activity. These results suggest that honey bees may be a suitable social animal model for future FASD research.

## 1. Introduction

The use of animal models for fetal alcohol spectrum disorder (FASD) research has contributed to our understanding of altered neural development in humans exposed to alcohol in utero [1,2]. While both vertebrate and invertebrate models of FASD effectively reproduce the clinical signs of FASD in humans, including decreased body weight, brain size, and developmental delay [3,4,5,6], few animal models have been successful in recapitulating the social behavioural effects of FASD, thereby limiting our understanding and treatment of these behaviours. Left untreated, the behavioural consequences of FASD are debilitating and increase the risk of addiction, mental illness, and incarceration in affected individuals [7].

Vertebrates, including fish, amphibians, and mammals, are widely used as animal models of FASD. Distinct advantages of the zebrafish *Danio rerio* and the clawed frog *Xenopus laevis* models of FASD include the low-cost production of large quantities of rapidly developing, easily visualized embryos that can be precisely exposed to ethanol in culture media to mirror many features of FASD in humans, such as abnormalities in craniofacial development and cognitive impairment [3,8,9]. Nevertheless, mammalian animal models, due to their more recent evolutionary relatedness to humans, are widely used in FASD research and present numerous scientific benefits. For example, mice offer the advantage of numerous transgenic lines, which can allow researchers to pinpoint specific developmental pathways altered by prenatal ethanol exposure [3,10]. Moreover, behavioural assays for motor skills and problem solving are well-developed in the rat model of FASD [3]. Guinea pigs are another promising model of FASD, particularly because the in utero stages of fetal brain development are closely aligned with similar neurodevelopmental stages in humans [3]. Primate models are considered the ‘gold standard’ for replicating the effects of FASD in humans, reflecting both human physiology and social behaviour; however, the high cost and ethical challenges often make primate studies unfeasible [3,11].

In addition to vertebrate models, invertebrate species such as *Caenorhabditis elegans* and *Drosophila melanogaster* have been increasingly utilized in FASD studies due to their low cost and ease of in vitro rearing as well as reduced regulatory requirements. For example, acute embryonic ethanol exposure of the nematode worm *C. elegans* produced physical abnormalities in cuticle morphology, while chronic larval ethanol exposure retarded growth and reproduction [12]. Furthermore, developmental ethanol exposure of *D. melanogaster* has been correlated with decreased insulin-like growth factor signalling and neuro proliferation and associated with teratogenic effects such as prolonged developmental time, decreased brain size, and altered tolerance to ethanol as adults [4,13,14].

Honey bees (*Apis mellifera*) share many of the same advantages as *Drosophila* as an insect research model [4,15,16,17,18], with the distinct advantage of highly social behaviour. Considering the highly tractable and sophisticated social behaviour of honey bees relative to other animal models of FASD, we suggest that honey bees may be a superior animal model for studying the behavioural effects of FASD in humans. Not surprisingly, honey bees have been proposed as models of complex human psychiatric conditions such as autism due to conserved genetic pathways for social responsiveness [18].

As a first step towards developing honey bees as an invertebrate model for the social behavioural effects of FASD, the purpose of this study was to determine if honey bees exposed to incremental concentrations of ethanol in the larval diet experience dose-dependent effects on developmental time, survival to adulthood, adult body weight, and motor activity similar to other animal models including *Drosophila*.

## 2. Materials and Methods

Honey bee larval rearing methodology was adapted from Schmehl et al. [15]. Control larvae were fed diets labelled ‘A’, ‘B’, and ‘C’, with increasing carbohydrate and protein concentrations (Supplementary Table S1), which were prepared using royal jelly (Stakich Inc., Troy, MI, USA), glucose (Fisher Chemicals, Fair Lawn, NJ, USA), fructose (Fisher Chemicals), yeast extract (Becton, Dickson, and Company, Franklin Lakes, NJ, USA), and distilled water. Ethanol-exposed larvae were reared on the same diets as controls, except that water was substituted with ethanol (Commercial Alcohols, Brampton, ON, CA) at three increasing concentrations (3, 6, and 10%) labelled ‘eA’, ‘eB’, and ‘eC’ (Table 1). Diets were stored at −20 °C until use.

Ethanol concentrations (3, 6, and 10%) were chosen based on their similarity to previously tested ethanol concentrations in other invertebrate models of FASD, including *D. melanogaster* (5, 10, and 12% EtOH) [4] and *C. elegans* (0.5 M = 3% EtOH) [12]. In *C. elegans*, exposure to 3% ethanol resulted in an internal alcohol concentration of approximately 120 mg/dL [12], which is within the range of the 100–400 mg/dL blood alcohol concentrations thought to induce FASD in humans and macaques [3,19,20].

To ensure the same age of experimental larvae, from mid-May until mid-August in 2022, mated queens were confined to an empty brood frame using a queen excluder cage within one of six well-established, healthy experimental field colonies. Twenty-four hours later, frames containing eggs were removed from the cage and maintained in a standard brood chamber in the same hive for three days. Subsequently, frames with newly hatched larvae were transported to the laboratory using a portable incubator at 35 °C.

Based on established protocols [15], within a biological safety cabinet, first instar larvae were grafted (transferred) from the brood frame into 48-well sterile tissue culture plates (STCP) containing 10 µL per well of control diet ‘A’ or ethanol-containing diet ‘eA’, pre-warmed to 35 °C. Each STCP was divided into three groups of 12 larvae, including one control group receiving an ethanol-free diet and two treatment groups receiving an ethanol-containing diet (3, 6, or 10% ethanol). To avoid ethanol vapour exposure of the controls, two empty vertical rows of wells of the STCP separated the control and treatment groups. A minimum of three technical replicates (*n* = 36 larvae) and four biological replicates (different queens representing different genetic lineages) were used for each group. After grafting, each larva received an additional 10 µL of control or ethanol-containing diet A and plates were incubated at 35 °C (mean  =  34.5 °C, SD  =  0.6) and 94% (mean  =  91.6%, SD  =  12.4) relative humidity.

Over the next five days, experimental larvae were fed increasing volumes of control diets ‘B’ or ‘C’ or ethanol-containing diets ‘eB’ and ‘eC’ in sequence, according to the protocol of Schmehl et al. [15]. On day six after grafting, larvae that consumed all diet were individually transferred to a new STCP (pupal STCP) and maintained at 35 °C (mean = 34.7%, SD = 0.3) and 75% (mean = 73.4, SD = 8.2) relative humidity until adult emergence. Larvae that did not consume all diet were maintained in the original STCP until all diet was consumed, or until death. The day of the transfer to the pupal STCP was recorded for each larva. Diet was not provided during pupation.

Larval and pupal survival was monitored daily by visual inspection with the aid of a dissecting microscope. Dead larvae and pupae were identified based on darkened coloration, deflation, and/or a lack of larval mobility and spiracle movement and were removed daily [15]. Then, 18–21 days after grafting, all live adult bees emerging from the pupal STCP were removed, chilled at −20 °C to immobilize, and weighed individually. Prior to chilling and weighing, four bees per group underwent locomotion analysis.

During locomotion analysis, the movement of an individual bee within a Petri dish was recorded for one minute using a high-definition Nikon D810 HD-SLR camera, ensuring consistent lighting conditions throughout the experiment. Subsequently, the locomotion activity trace and total distance travelled (cm) were determined using the online motion-tracking software BehaviorCloud (https://www.behaviorcloud.com/ (accessed on 8 September 2022), San Diego, CA, USA).

Statistical analysis was conductedusing R Statistical Software (v4.2.3; R Core Team 2023). Data for the day of onset of pupation data were non-normally distributed (Shapiro–Wilk, W = 0.61; *p* < 0.001), and treatments and controls were compared using a Kruskal–Wallis test and a post hoc Dunn’s test. Data for total body weight and distance travelled were normally distributed (Shapiro–Wilk, W = 0.99 and W = 0.96, respectively; *p* > 0.05 for both), and treatment and control groups were compared using a one-way analysis of variance (ANOVA), followed by a post hoc Tukey’s test. Additionally, survival analysis was carried out using the Mantel–Cox log-rank test.

## 3. Results

### 3.1. Survival Analysis

**Figure 1:**
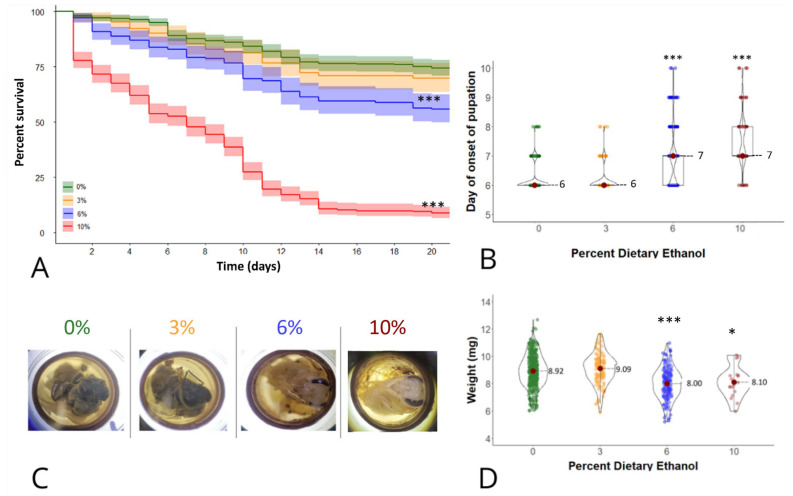
Effects of larval ethanol exposure on honey bee development in vitro. (**A**) Lines represent mean survival with a 95% confidence interval indicated by shading of 588, 192, 240, and 480 larvae exposed to 0, 3, 6 and 10% ethanol, respectively, in the larval diet. *** indicates a significant difference from the control with *p* < 0.001 by a Mantel–Cox log-rank test. (**B**) Data points represent the day of onset of pupation of 566, 189, 237, and 103 individual larvae. The width of each curve of the violin corresponds to the approximate frequency of data points in each region. The median and interquartile range are indicated for each group. *** indicates a significant difference from the control with *p*  <  0.001 by a Kruskal–Wallis and Dunn’s test. (**C**) Representative, age-matched honey bees at day 18 of in vitro rearing after receiving a larval diet containing 0%, 3%, 6%, or 10% ethanol. The gross morphology of the 0% and 3% groups is consistent with an imago, while the morphology of the 6% and 10% groups is consistent with a pre-imago based on the extent of cuticular pigmentation. (**D**) Data points represent body weight in milligrams (mg) of 437, 134, 134, and 18 individual adult bees surviving to eclosion after exposure to 0%, 3%, 6%, or 10% ethanol, respectively. The width of each curve of the violin corresponds to the approximate frequency of data points in each region. The mean and standard deviation are indicated for each group. *** and * indicate significant differences from the control with *p* < 0.001 and *p*  <  0.05, respectively, by one-way ANOVA and Tukey’s test.

Repeated honey bee larval exposure to ethanol on days 1, 2, 3, 4, and 5 (post-hatching) resulted in a dose-dependent decrease in survival. Notably, larvae reared on a diet containing 10% ethanol exhibited, on average, a significant mean 45% reduction (mean percent survival = 52%, 95% CI: 48–57%) in survival during larval development (until pupation/day 6) compared to the control group (Figure 1A; Log-rank test, X^2^ (1) = 43.5, *p* < 0.001). Additionally, when assessing survival to adult emergence (until day 21), larvae reared on diets containing 6 and 10% ethanol displayed a mean 20% reduction in survival (mean percent survival = 55%, 95% CI: 49–62%) and a mean 71% reduction in survival (mean percent survival = 6.5%, 95% CI: 6–11%), respectively, in contrast to the control group (Log-rank test, X^2^ (1) = 43.5, *p* < 0.001 for 6% and X^2^ (1) = 709, *p* < 0.001 for 10% group; Figure 1A).

### 3.2. Developmental Delays

In addition to decreased survival, chronic honey bee larval ethanol exposure was characterized by significant developmental delays (Figure 1B,C). Larvae exposed to 6% and 10% dietary ethanol experienced a significant, one-day delay (median = 7 for both groups; 95% CI = 6–7 for 6% group and 95% CI = 7–8 for 10% group) in completing larval development relative to control larvae (Figure 1B; Kruskall Wallis, X^2^ (3) = 398.5, post hoc Dunn’s test *p* < 0.001 for both 6% ethanol and 10% groups). Moreover, a gross, qualitative evaluation of the experimental bees on day 18 of in vitro rearing (Figure 1C) revealed that the bees exposed to 0% and 3% ethanol exhibited the expected morphological characteristics of an imago, including complete development and full body pigmentation [21]. In contrast, the groups exposed to 6% and 10% ethanol appeared to be in the pre-imago stage, showing complete body development but a lack of cuticular pigmentation [21].

### 3.3. Weight at Eclosion

Developmental ethanol exposure of honey bees was associated with decreased weight at eclosion (birth). Adult bees reared on a 6% and 10% ethanol-containing diet demonstrated, on average, a significant 10% (mean = 0.92 ± SD = 0.01 mg and 0.82 ± 0.01 mg, respectively), reduction in mean body weight relative to controls (Figure 1D; ANOVA, F_3,819_ = 33.2, Tukey’s test *p* < 0.001 for 6% group and *p* = 0.02 for 10% group).

### 3.4. Locomotion Analysis

**Figure 2:**
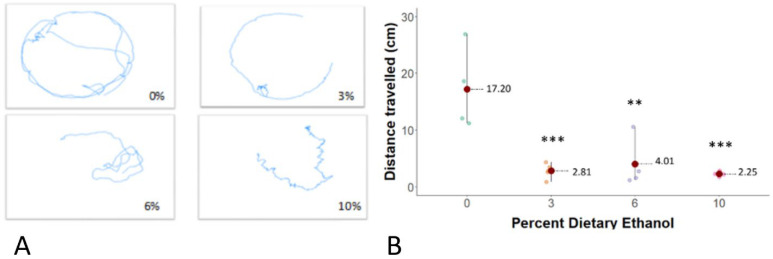
Reduction in locomotion of ethanol-reared adult bees. (**A**) Representative movement tracing over one minute (BehaviorCloud^©^, Colombus, OH, USA) of one newly emerged adult bee per group in a Petri dish. (**B**) Dots represent distance travelled in centimetres during one minute by one adult bee, with the mean and standard derivation indicated for each group. *** and ** indicates significant differences with *p* ≤ 0.001 and *p* < 0.01 by one-way ANOVA and a Tukey’s test.

We found that bees exposed to ethanol during development had decreased mobility as adults, as evidenced by a significant mean 12 ± SD = 1.3 cm to 14 ± 4.4 cm decrease in distance travelled for the 3, 6, and 9% groups, respectively, compared to controls (Figure 2B; one-way ANOVA, F_3,12_ =12.1; Tukey’s test *p* < 0.001 for 3% group; *p* = 0.003 for 6% group; *p* = 0.001 for 10% group).

## 4. Discussion

Using a novel honey bee animal model, we successfully recapitulated many of the features of developmental ethanol exposure reported in other animal model species of FASD, including dose-responsive effects on survival, developmental time, and body weight, as well as deficits in adult locomotion. The observed dose-dependent decrease in survival among honey bee larvae reared on ethanol is consistent with findings conducted on a *Drosophila* model of FASD [4]. In particular, flies exposed to 10% ethanol during development showed a significant 60% decrease in survival to adulthood [4]. Similarly, we found that honey bee larvae raised on diets containing 6% and 10% ethanol experienced significant, 20% and 70% decreases in survival to adult eclosion, respectively (Figure 1A).

Developmental delay has been previously reported as a feature of prenatal ethanol exposure in other animal models, including *Drosophila* [4], rats [5], and zebrafish [6]. Typically, honey bee pupae complete their body development and emerge as imagos on the seventeenth day post-hatching [21]. Our gross evaluation of in vitro pupal development (Figure 1C), however, showed that larvae reared on 6 and 10% ethanol do not show the expected morphology of an imago, based on incomplete pigmentation of the body, aligning with the one-day delay in larval development to pupation reported for the same treatments.

Similar to humans [22] and other animal models of FASD [4,23,24], developmental ethanol exposure of honey bees was associated with decreased weight at eclosion (birth). Corresponding effects have been observed in *Drosophila*, with an approximate 20% reduction in body weight when exposed to diets containing 5 and 10% ethanol [4]. Similarly, in rats, there was an approximate 12% decrease in body weight at birth when dams consumed 30% of ethanol in water solution during the gestation [23], while in mice, an 11% decrease in birthweight was observed when dams consumed 25% ethanol in water solution ad libitum during gestation [25]. While the possibility remains that observed effects of larval ethanol exposure on adult body weight could be linked to suboptimal nutrition resulting from altered larval food consumption, we ensured complete consumption of all larval diets prior to transferring larvae to begin pupation. Therefore, any decrease in body weight at eclosion was most likely related to the direct effects of ethanol exposure. Moreover, in previous work, diets containing 5% ethanol were not shown to affect the food consumption of *Drosophila* larvae [4]. The observed decrease in locomotion of adult bees exposed to ethanol as larvae suggests that developmental ethanol exposure induces long-lasting neurobehavioural effects in exposed bees. Despite a limited sample size (*n* = 4 per group), our findings are consistent with previous studies demonstrating the adverse effects of developmental ethanol exposure on motor coordination and reflex development in rats [26] and swimming activity in zebrafish [27]. Similarly, in primates, the consumption of high doses of ethanol during gestation resulted in decreased motor activity in infants [19]. In contrast, developmental ethanol exposure in *Drosophila* was associated with increased locomotor activity in response to ethanol vapours as adults [4]. Importantly, the observed deficits in locomotion in ethanol-reared bees in this study suggest alterations in neural development, which may have sociobehavioural consequences. Future directions of this research may include mechanistic investigation of the neurochemical and neurodevelopmental pathways altered by developmental ethanol exposure of honey bees using RNA sequencing and gene expression studies.

Humans affected by FASD often experience deficits in social skills, including impaired communication, reduced empathy, poor social judgement and problem-solving, inappropriate social behaviour, and decreased ability to establish and maintain healthy peer and family relationships [28]. In turn, social skill deficits can predispose individuals with FASD to other secondary behavioural problems and disabilities, such as aggression, hyperactivity, depression, anxiety, and addiction [28]. By comparison, a suite of normal [29,30] social behaviours, such as waggle dance communication, as well as abnormal social behaviours [18], such as lack of nursing and guarding behaviour, have been reported, tracked and quantified in honey bees [18]; accordingly, our future studies will test the hypothesis that honey bees exposed to incremental doses of ethanol during the development will exhibit incremental social behavioural disorders, similar to those observed in humans affected by FASD. Providing that our hypothesis is not rejected, the honey bee may emerge as a superior animal model for behavioural FASD research that could be comparable to non-human primate FASD models but with substantially lower costs and fewer bioethical concerns.

## 5. Conclusions

In this study, developmental ethanol exposure of honey bee larvae reduced survival to adult eclosion, delayed development, and decreased body weight and locomotion of adult bees, similar to other animal models of FASD. Taken together, these ethanol-associated changes suggest honey bees are a suitable social animal model for FASD research.

## Figures and Tables

**Table 1:** Ethanol-containing diets used for in vitro larval rearing. Volumes of ethanol (mL) are indicated for each percent ethanol concentration, with total diet volume in ml indicated in parentheses.

% EtOH in Diet	Diet eA (9.1 mL)	Diet eB (9.1 mL)	Diet eC (45.4 mL)
3	0.29	0.29	1.44
6	0.57	0.57	2.89
10	0.87	0.87	4.34

## Data Availability

All data and R scripts used in this study are available in the GitHub repository: https://github.com/mcamilli/LifeFASDData (accessed on 21 March 2024).

## Supplementary Materials

The following supporting information can be downloaded at https://www.mdpi.com/article/10.3390/life14040434/s1. Table S1: Amount and percentage of diet components in the larval diet necessary to feed approximately 400 larvae reproduced from Schmehl et al. [15].
