# Supplementary material for: Fetal Alcohol Spectrum Disorder: The Honey Bee as a Social Animal Model
Source: Life (Basel). 2024 Mar 25;14(4):434. doi: 10.3390/life14040434 (PMC11051024; doi:10.3390/life14040434)
Supplement: Supplementary file 1 [file life-14-00434-s001.zip › life-2915263-SI.pdf]

TableS1. Amount and percentage of diet components in the larval diet necessary to feed approximately 400 larvae according to Schmehl et al. [15].

	Amount of diet components (g)			Percentage of diet components in total diet		
**Diet Component**	**Diet A**	**Diet B**	**Diet C**	**Diet A**	**Diet B**	**Diet C**
**Royal jelly**	4.43	4.30	25.00	44.25	42.95	50.00
**Glucose**	0.53	0.64	4.50	5.30	6.40	9.00
**Fructose**	0.53	0.64	4.50	5.30	6.40	9.00
**Yeast extract**	0.09	0.13	1.00	0.90	1.30	2.00
**Water**	4.43	4.30	15.00	44.25	42.95	30.00
**Total**	10	10	50	100	100	100
